# Monitoring of a microbial community during bioaugmentation with hydrogenotrophic methanogens to improve methane yield of an anaerobic digestion process

**DOI:** 10.1007/s10529-023-03414-7

**Published:** 2023-08-03

**Authors:** Aixa Kari Gállego-Bravo, Jaime García-Mena, Alberto Piña-Escobedo, Gloria López-Jiménez, María Eugenia Gutiérrez-Castillo, Luis Raúl Tovar-Gálvez

**Affiliations:** 1 Instituto Politécnico Nacional, Centro Mexicano Para la Producción más Limpia, Av. Acueducto s/n, 07340 Ciudad de Mexico, Mexico; 2 Departamento de Genética y Biología Molecular, Cinvestav, Av. Instituto Politécnico Nacional 2508, 07360 Ciudad de México, Mexico; 3 Departamento de Ciencias Básicas, Instituto Politécnico Nacional, Unidad Profesional Interdisciplinaria de Biotecnología, Av. Acueducto s/n, 07340 Ciudad de Mexico, Mexico; 4 Instituto Politécnico Nacional, Centro Interdisciplinario de Investigaciones y Estudios Sobre Medio Ambiente y Desarrollo, Calle 30 de Junio de 1520 s/n, 07340 Ciudad de Mexico, Mexico

**Keywords:** Anaerobic digestion, Bioaugmentation, Hydrogenotrophic methanogens, Methane yield, OFMSW

## Abstract

Methane production by microbial fermentation of municipal waste is a challenge for better yield processes. This work describes the characterization of a hydrogenotrophic methanogen microbial community used in a bioaugmentation procedure to improve the methane yield in a thermophilic anaerobic process, digesting the organic fraction of municipal solid waste. The performance of the bioaugmentation was assessed in terms of methane production and changes in the microbial community structure. The results showed that bioaugmentation slightly improved the cumulative methane yield (+ 4%) in comparison to the control, and its use led to an acceleration of the methanogenesis stage. We observed associated significant changes in the relative abundance of taxa and their interactions, using high throughput DNA sequencing of V3-16S rRNA gene libraries, where the abundance of the archaeal hydrogenotrophic genus *Methanoculleus* (class Methanomicrobia, phylum Euryarchaeota) and the bacterial order MBA08 (class Clostridia, phylum Firmicutes) were dominant. The relevant predicted metabolic pathways agreed with substrate degradation and the anaerobic methanogenic process. The purpose of the study was to evaluate the effect of the addition of hydrogenotrophic methanogens in the generation of methane, while treating organic waste through anaerobic digestion.

**Supplementary Information:**

The online version contains supplementary material available at 10.1007/s10529-023-03414-7.

## Introduction

Anaerobic digestion (AD) is used for the treatment of organic waste, with the benefit of avoiding water, air, and soil contamination due to the poor removal of organic wastes. AD also produces sustainable energy like biogas and digestate as a soil amendment or fertilizer (Bong et al. 2018).

Although AD has been extensively studied, current research focuses on the improvement of the process, through various techniques such as co-digestion (Seruga et al. 2018), pretreatments such as physical, chemical, mechanical, and/or biological (Liu et al. 2020), CO_2_ bio-sequestration (Muntau et al. 2021; Xu et al. 2021), reactor configurations (Maspolim et al. 2015), additive applications (Barua et al. 2019), bioaugmentation (Ariunbaatar et al. 2017; Lianhua et al. 2018), or a combination of all of them (Mulat et al. 2018). The bioaugmentation technique is defined as the use of microorganisms grown independently (pure cultures, defined mixed cultures, or consortia) which are added to a biological system to improve the process (Fotidis et al. 2014; Lebiocka et al. 2018). This approach has been used for several purposes relieving overloaded anaerobic digesters (Tale et al. 2011; Li et al. 2018), alleviating ammonia inhibition and salinity stress (Wang et al. 2023; Duc et al. 2023), and enhancing methane production (Zhang et al. 2015; Aydin 2016; Strang et al. 2017).

To increase methane yield, several reported technologies have used bioaugmentation employing different types of microorganisms. One report used a proprietary cellulolytic culture to raise the hydrolysis rate from sweet corn wastes increasing the efficiency of methanogenesis in a two-phase AD process, resulting in a methane raise of 56% in comparison with the non-bioaugmentation reactor (Martin-Ryals et al. 2015). Another report used *Clostridium thermocellum* in batch reactors packed with agricultural wastes (Tsapekos et al. 2017), showing a methane enhancement of 34%; a similar result was achieved with a continuously stirred tank reactor (CSTRs); however, at steady state, the outcome was less efficient. Another work treating potato juice, used axenic methanogenic cultures of *Methanothermobacter thermautotrophicus* and *Methanosarcina termophila* to improve the methane yield by 40% in an up-flow anaerobic sludge blanket (UASB) reactor from mesophilic to thermophilic conditions (Zhu et al. 2018). In another example, a system with high ammonia content (cattle manure and microalgae) employed *Methanoculleus bourgensis,* and the methane production increased by 28% after bioaugmentation (Tian et al. 2019).

A leachate, like the one harvested from the composting Plant “Bordo Poniente” (CPBP) in Mexico City (GPS coordinates 19.48035—98.97206), can be employed to generate an active acclimated-inoculum, used as a startup of AD of organic fractions of municipal solid waste (OFMSW) due to its advantageous microbial composition (Gállego Bravo et al. 2019). In this work, the content of hydrogenotrophic methanogens was enriched and reintegrated into the system, as part of the bioaugmentation technique, to improve the methane yield of a thermophilic anaerobic digestion process treating OFMSW. To better understand the process, the bacterial and archaeal microbial diversity was characterized in the acclimated inoculum, the methanogenic consortium, and the bioaugmentation process.

## Materials and methods

### Substrate and inoculum sampling

The organic fraction of municipal solid waste (OFMSW) and the leachate used as inoculum, were both harvested in the CPBP on the outskirts of Mexico City. Approximately 6 L of leachate was collected, while the OFMSW was sampled using the quarter method for solid waste samples, and ~ 2 kg fractions were obtained. All samples were transported to the laboratory within 30 min time distance from the CPBP; the leachate samples were stored at 55 °C for 2 weeks (CPBP inoculum), while the OFMSW was chopped down to ~ 1 cm^3^ fragments and stored at −20 °C until use.

### Preparation of methanogenic consortium

The initial hydrogenotrophic methanogen consortium was prepared using the CPBP inoculum. The medium for isolation (MI) had the following composition: 1.0 g NH_4_Cl, 0.3 g KH_2_PO_4_, 0.3 g K_2_HPO_4_, 0.2 g MgCl_2_·6H_2_O, 2.0 g NaCl, 0.1 g CaCl_2_·2H_2_O, 0.1 g KCl, 0.5 g cysteine-HCl, 1.0 g yeast extract, 1.0 g peptone, 10 mL trace element solution and 1 mL resazurin in 1 L final volume of distilled water. The content of the trace element solution per 1 L final volume in distilled water was: 1.5 g nitrilotriacetic acid, 2.5 g MgCl_2_·6H_2_O, 0.6 g MnCl_2_·4H_2_O, 1.0 g NaCl, 0.1 FeCl·4H_2_O, 0.1 g CoCl_2_·6H_2_O, 0.01 g AlCl_3_, 0.01 g H_3_BO_3_, 0.01 g Na_2_MoO_4_·2H_2_O, 0.001 CuCl_2_·2H_2_O, 0.1 g CaCl_2_·2H_2_O and 0.1 g ZnCl_2_. The MI was sterilized at 121° C for 15 min and the pH was adjusted to 7.0 with 10% autoclaved NaHCO_3_ under N_2_ gassing. 5 mL of the CPBP–inoculum was added to 45 mL of sterilized medium and immediately flushed with H_2_/CO_2_ (80%/20%) at 20 psi for 3 min. The mixture was incubated at 55 °C for 4 weeks to obtain the initial hydrogenotrophic methanogen consortium (iMC). To generate the hydrogenotrophic methanogenic consortium (MC) a 5 mL aliquot of iMC was added to 45 mL fresh MI. Every time a new methanogenic consortium was needed, 5 mL of the previous MC was added to 45 mL of fresh MI. To verify that the MC had methanogens, the methane content was determined by gas chromatography as described in the Analytical methods section.

### Experimental setup

The biochemical methane potential test (BMP) was used in batch mode to determine the effect of bioaugmentation on methane yields. The BMPs were loaded in 125 mL serum bottles filled with a substrate/inoculum ratio of 1:1 volatile solid (VS) in a working volume of 60 mL. The bottles were tightly capped with butyl rubber stoppers and sealed with aluminum crimps. The air from the serum bottles was drawn with a needle–syringe and replaced with flushing helium for 20 s (20 psi). The treatments were incubated at 55° C and shaken at 60 rpm for 42 days. The controls were AI100 (CPBP inoculum + water), and MC00 (inoculum + OFMSW). The BMPs reactors consisted of OFMSW in MC10 (90% inoculum + 10% methanogenic consortium (MC) v/v), MC25 (75% inoculum + 25% MC v/v), MC50 (50% inoculum + 50% MC v/v), and MC75 (25% inoculum + 75% MC v/v). The MC was injected using sterile syringes into the closed serum bottles at the start of the experiment. The value for methane production of the negative control (AI100) was subtracted from the methane generated on each treatment.

### Analytical methods

Total solids (TS) and volatile solids (VS) were determined by gravimetric methods. The samples for TS were dried at 70 °C for 24 h, while the samples for VS were calcined at 550 °C for 2 h. The pH and oxidation–reduction potential (ORP) were measured using a Hanna Instruments pH meter model HI98191, and the electrical conductivity (EC) was measured using a Hanna Instruments electrode model HI99300. The methane (CH_4_) and carbon dioxide (CO_2_) content were analyzed using a gas chromatograph (Perkin Elmer Autosystem, Waltham, MA, USA), equipped with a thermal conductivity detector and a Porapack column (QS SS 80/100 12’ × 1/8’’ × 0.085’’, Alltech). The generation of biogas was evaluated by water displacement using graduated cylinders (Martin-Ryals et al. 2015). All tests were performed in triplicate. For physicochemical analyses, samples were taken at the beginning and end of the experiment, for the microbial communities on days 0, 17, and 32, and composition and generation of biogas every 2–3 days.

### High-throughput DNA sequencing

Genomic DNA was extracted from 400 µL of the methanogenic consortium, all BMP treatment groups, or 400 µg of the OFMSW, using the DNeasy PowerLyzer PowerSoil Kit 100 (QIAGEN, Germany, Cat# 12855–100) according to the manufacturer’s instructions. The preparation of 16S rDNA gene libraries (bacteria and archaea) and the high throughput sequencing by the Ion Torrent PGM system was followed as previously described (Gállego Bravo et al. 2019). Sequences were processed by QIIME 2 pipeline (v 2019.10). Identified features were picked up against the Greengenes (v13.8) database at 99% similarity obtaining the feature-table.biom and dna-sequences.fasta files.

### Bioinformatic analyses

Alpha diversity, including the Observed number of species, Chao1, Shannon, and Simpson indexes, was calculated using phyloseq (v1.22.3) package in R (v3.4.4), Python 3.0 and Jupyter Notebook software; while the beta diversity was measured using UniFrac analysis and plotted by principal coordinate analysis (PCoA). Taxa with relative abundances significantly different among treatments were determined by linear discriminant effect size analysis using LefSe (v1.0) or One-way ANOVA and post-hoc analysis. The prediction of the metabolic pathways was determined using PICRUSt2 (v01) (Douglas et al. 2020), and the KEGG (Kyoto Encyclopedia of Genes and Genomes) database. The STAMP (v2.1.3) was used for statistical analysis and visualization. Co-occurrence analysis was done through the web-based tool MicrobiomeAnalyst (Chong et al. 2020).

### Statistical analyses

For the methane yields, chemical analysis data, and alpha diversity values One-way ANOVA was done using SigmaPlot (v12.0) software; for beta diversity, PERMANOVA analysis was performed; Benjamini–Hochberg correction was used to determine the false discovery rate error in PICRUSt2. For the co-occurrence analysis, Pearson’s correlation was made. Values of *p* < 0.05 and *q* < 0.05 were considered statistically significant.

## Results and discussion

### Effect of bioaugmentation with hydrogenotrophic methanogen on reactor performance

**Fig. 1:**
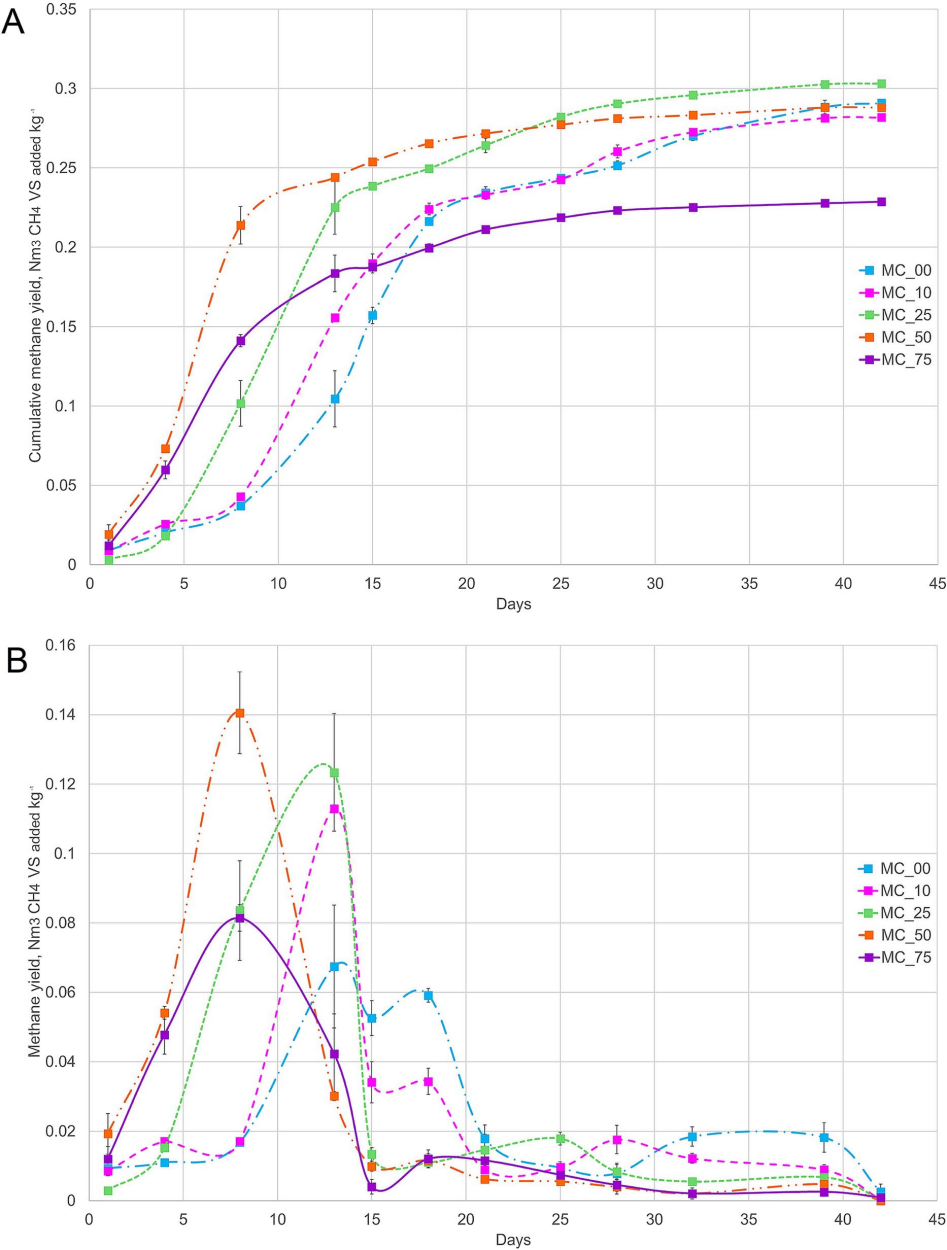
Reactor performance during the thermophilic anaerobic digestion over time showing **a** cumulative methane yield and **b** daily methane yield. The long–dash–dot light blue line shows the methanogenic consortium at 0% (MC00); the dashed fuchsia line shows the methanogenic consortium at 10% (MC10); the square–dot green line shows the methanogenic consortium at 25% (MC25); the long–dash–dot–dot orange line shows the methanogenic consortium at 50% (MC50); and the solid purple line shows the methanogenic consortium at 75% (MC75). Y-axis indicates the methane yield expressed as m^3^ CH_4_ VS_added_ kg^−1^, and the X-axis indicates the time in days. Tags with filled squares at the right side of each graph show the different treatments. The experiments were done in triplicate and values are shown as mean ± standard deviation at each point in the graphics

The measurement of the cumulative yield showed that all treatment reactors accumulated biogas during the 42 days, reaching values from 0.369 ± 0.061 to 0.488 ± 0.020 m^3^ VS_added_ kg^−1^, depending on the percentage of the methanogenic consortium used. The methane content in the biogas increased slowly over time, achieving values from 61% to 79%, leading to a cumulative methane yield from 0.229 ± 0.028 to 0.303 ± 0.007 m^3^ CH_4_ VS_added_ kg^−1^ (Fig. 1A). The reactor MC25 got the highest cumulative methane yield (0.303 ± 0.007 m^3^ CH_4_ VS_added_ kg^−1^) followed by MC00 (non-bioaugmented) and MC50.

A characteristic that stood out in the bioaugmented reactors was the acceleration of methanogenesis in comparison with the control (Fig. 1B). The MC50 condition had the highest peak in the shortest time (day 8), with a methane content of 75.03%, followed by MC25 which had its peak at day 13 with a methane content of 79.03%. MC00 had 2 peaks, one on day 13 and the other on day 18, with a methane content of 68.53% and 76.50%, respectively. When comparing the yield of the bioaugmented treatments with the MC00 control, there was an increase in the biogas of 3.39% and the methane of 4.62%, but no significant (p > 0.05) differences were found. Even if the methane increase was not statistically significant (p > 0.05), the addition of MC caused the methane generation raised earlier (by volume), with better biogas quality compared to the control. These results are relevant to decrease the retention time of any given substrate in anaerobic digesters (Akyol et al. 2019).

At the end of the digestion process, the physicochemical characteristics (Table S1) showed that the pH remained close to neutral values; ORP indicates that anaerobic conditions were maintained, however, in the case of MC75 a positive value was obtained, suggesting aerobic respiration occurred, and therefore the generation of methane could be affected (Gerardi 2003). In terms of VS reduction, MC75 got the highest efficiency where a maximum of 73.38% was achieved, followed by MC10 (72.50%), MC50 (71.10%), MC25 (67.95%), and finally MC00 (63.12%).

### Microbial succession in the methanogenic consortium during OFMSW degradation

**Fig. 2:**
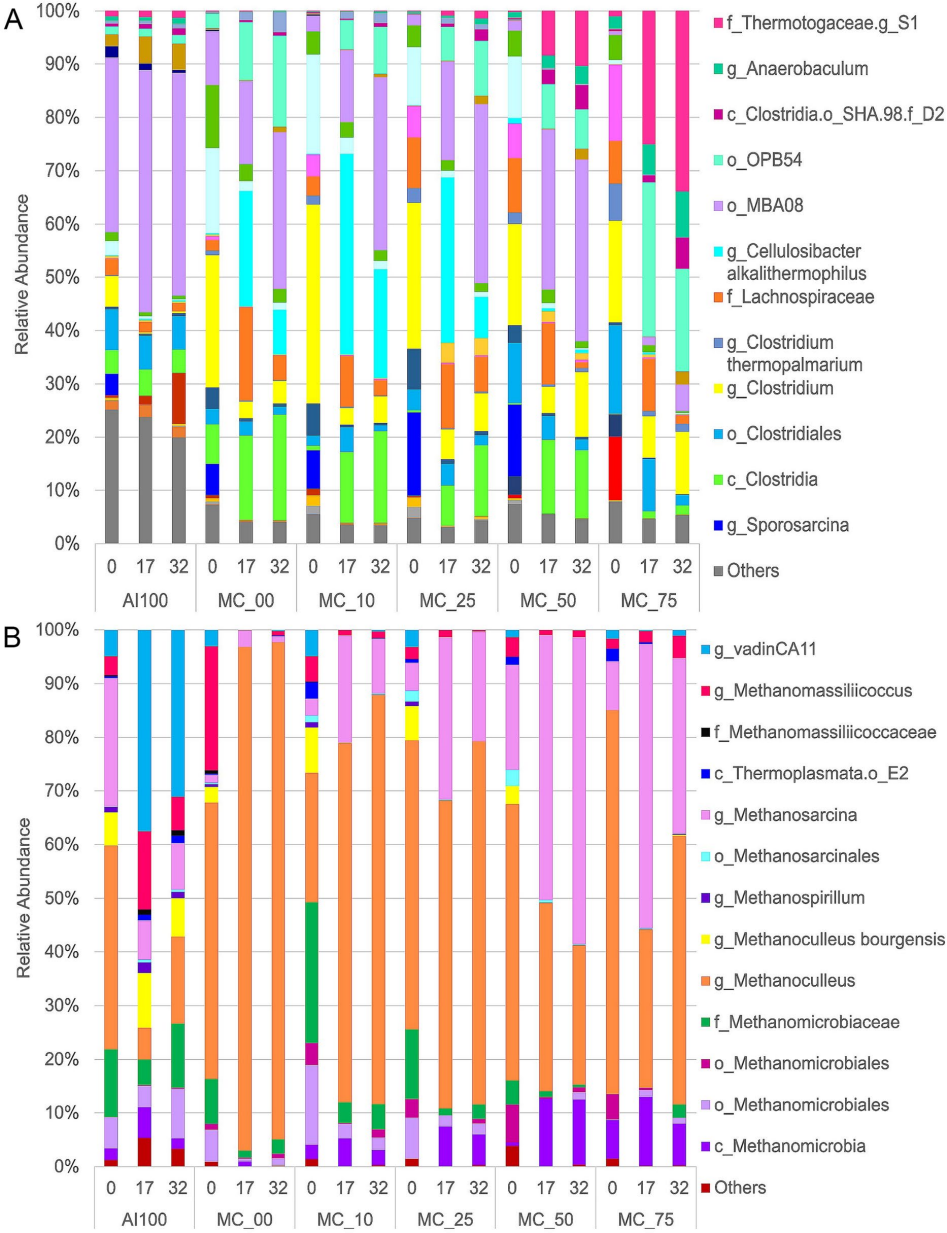
Relative abundance of predominant bacterial and archaeal taxa in batch thermophilic anaerobic digesters. The stacked bar charts show abundances for **a** bacterial class, order, family, genera, or species, **b** archaeal class, order, family, genera, or species, for the methanogenic consortium at 0% (MC00), 10% (MC10), 25% (MC25); 50% (MC50), 75% (MC75), and the negative control for the process (AI100). Y-axis indicates the percentage of relative abundance; X-axis indicates the time in days and treatment. Tags at the right side of each set of graphic bars identify the corresponding taxa by color. Plotted data are the average of three independent sequencing experiments

The high throughput DNA sequencing of V3-16S rDNA libraries for bacteria produced a minimum of 14,706 and a maximum of 101,920 reads with an average of 45,062.94 (Table S2). The data analyses at the phylum level showed a dominance of Firmicutes except for the MC50 and MC75 where an increase of the Thermotogae and Synergistetes phyla were observed (Fig. S4A). When the relative abundance of other taxa was characterized, members of the order MBA08 (class Clostridia, phylum Firmicutes), were remarkably enriched at day 17 and day 32 in the MC50 reactor and in most of the treatments (Fig. 2A). The predominant phyla in MC were Synergistetes and Firmicutes, while in the OFMSW the phylum Proteobacteria was predominant (Fig. S2). Synergistetes is known to produce acetic acid and hydrogen while Thermotogae degrade acetate, both phyla collaborate syntrophically with hydrogenotrophic methanogens (Ferguson et al. 2018; Xu et al. 2021).

The alpha diversity indexes (Observed, Chao1, and Shannon) had the highest values for AI100 and the lowest for MC75. In general, a decreasing tendency was observed for the diversity values along the experimental time (Fig. S5A-C, Table S3). Regarding the Simpson index, the MC10 treatment started with the lowest values at day 0 and increased through time up to day 32, while the converse was observed for AI100, MC50, and MC75 (Fig. S5D, Table S3). Overall, the observed number of species, and the richness decreased during the experiment; this was shown as lower diversity in the bacterial community as the indices suggest. In agreement with our results, a work of fungal bioaugmentation with lignocellulosic biomass, reported higher alpha diversity values in the control reactors than in those that were bioaugmented (Akyol et al. 2019); one more report of a methanogenic process reported a decreasing number of observed OUT in their bioaugmented system as well as an increase in the organic loading rate (OLR) (Lianhua et al. 2018).

**Fig. 3:**
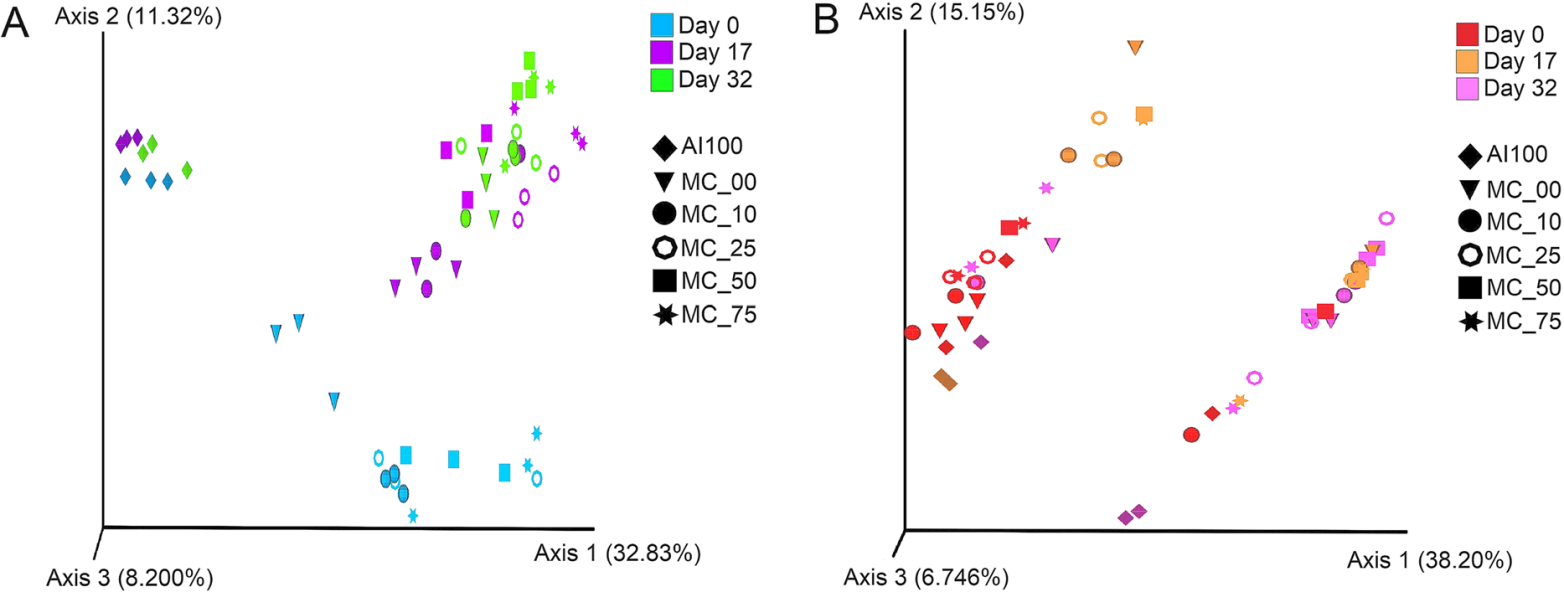
Microbiota diversity during the thermophilic anaerobic digestion over time. The figure shows beta-diversity analyses of bacterial **a** and archaeal **b** communities. Dissimilarity metrics were calculated by Unweighted UniFrac analysis. Three-dimensional scatter plots were generated using principal coordinates analysis (PCoA) in three different axes, which shows the percentage of total differences. *p* values were calculated using the PERMANOVA test to compare distance among groups. Tags at the right upper part beside each plot indicate time in days 0 (bacteria: light blue; archaea: red), 17 (bacteria: purple; archaea: orange), and 32 (bacteria: green; archaea: pink). Black and white symbols identify each methanogenic consortium concentration, black color inverted triangle (0%, MC00), black color filled circle (10%, MC10), black color ring (25%, MC25); black color filled square (50%, MC50), black color filled star (75%, MC75), and black filled diamonds, the negative control for the process (AI100)

Bacterial beta diversity showed different clusters between AI100 and the rest of the treatments, where there were detectable changes between day 0 to day 17 and less noticeable changes around day 32. Treatment MC00 clustered separately from the other groups, being significantly different from MC25, MC50, and MC75 (Fig. 3A, Table S4). AI100 beta diversity barely changed and the values at days 0, 17, and 32 were clustered (Fig. 3A, Table S4). The addition of the OFMSW to the acclimated inoculum (MC00), provided sufficient substrate to promote the proliferation of the bacterial community. The same occurred when increasing amounts of the methanogenic consortium were added from 10 to 75% (Fig. 3A, Table S4). Similarly, two different reports using enriched cultures reported clear separations between the control reactors and those that were bioaugmented either with fungal or methanogenic cultures (Akyol et al. 2019; Xu et al. 2021).

The sequencing for archaea produced a minimum of 1,051 and a maximum of 33,556 reads with an average of 11,179.92 (Table S2). The analyses of the data at the class level showed a dominance of Methanomicrobia except for the AI100 where an increase of the class Thermoplasmata was observed (Fig. S4B). When the relative abundance of other taxa was characterized, members of the genus *Methanoculleus* were remarkably enriched in MC00 (Fig. 2B). The abundance of this taxa decreased with days of fermentation, when the MC concentration increased up to 75%, along with an increase of *Methanosarcina*. This was notoriously observed in MC50, the condition with methane production in the shortest time (Fig. 2B). The genus vadinCA11 from the family Methanomassillicoccaceae predominated only in AI100. The archaeal composition of MC revealed that it mostly contained the genus *Methanoculleus* (Fig. S3). This species is widely known to belong to hydrogenotrophic methanogens while *Methanosarcina* is a mixotrophic methanogen, since it can use acetate, hydrogen, CO2, methanol, and methylated amines to produce methane (Oosterkamp et al. 2019).

The alpha diversity indexes showed different trends in all treatments. The richness indexes Observed and Chao1 increased in treatments AI100 and MC75 through time and decreased in MC50. In the rest of the treatments, the values decreased on day 17 and increased on day 32 (Fig. S5E-F, Table S3). Regarding the Shannon index, the diversity values increased in treatments AI100 and MC75 during the process and decreased in the rest of them. A similar trend was observed for Simpson index values, however, in MC25 there was an increase from day 0 to day 17 and a decay at day 32 (Fig. S5G-H, Table S3).

On the other hand, the Archaeal beta diversity displayed two evident clusters, though without any clear trend in time or treatments. In one of the clusters, samples from day 0 and day 17 were grouped, while the other cluster grouped those of day 32 (Fig. 3B, Table S4). The bacterial and archaeal alpha diversity indexes indicate that, in general, as the volume of MC increased, the richness and diversity of the microbial community decreased, which could have a direct impact on the generation of methane, however, in the case of MC50, the values were downward through time. Unfortunately, it was not possible to collect the sample at the precise methane peak to know the microbial composition and diversity.

### Significant changes in taxa abundance and microbial interactions

**Fig. 4:**
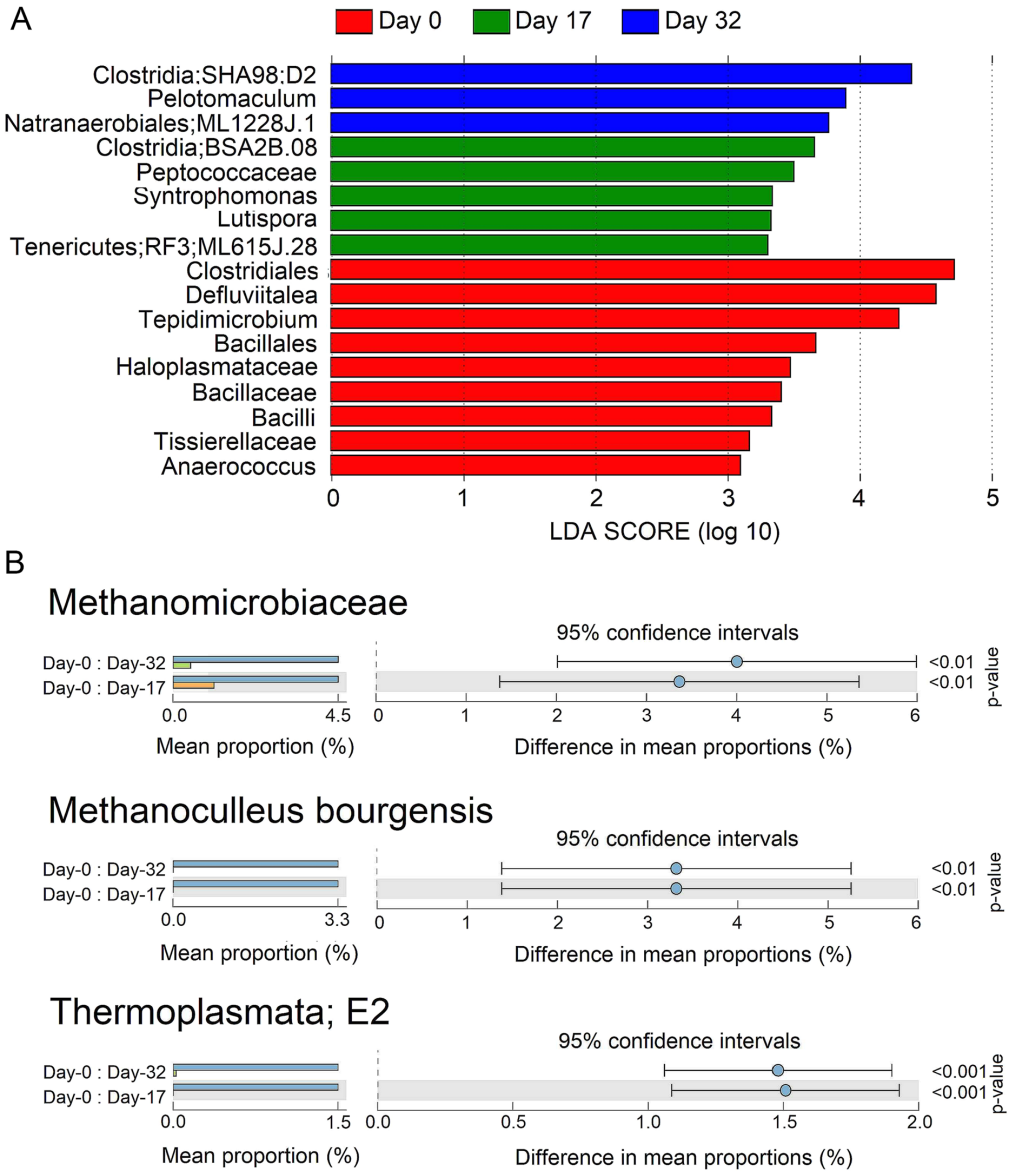
Differentially abundant taxa and their occurrence for the MC50 treatment. Linear discriminant analysis (LDA) effect size (LEfSe) for bacteria. Horizontal bars represent the effect size for each taxon. For all shown taxa, LDA score > 3.0, and *p* < 0.05 (**a**). One-way ANOVA for archaeal taxa, *p* < 0.05 (**b**)

We analyzed the changes in the abundance of specific taxa and their interactions in the community in all experimental conditions MC00, MC10, MC25, MC50, and MC75. From all these, we chose the MC50 treatment since this condition exhibited the highest methane production at the earliest time (Fig. 1B). The analysis of the bacterial abundance based on the effect size using the LefSe software for MC50 treatment, revealed nine different abundant bacterial taxa at day-0, five for day-17, and three for day-32. Most of these taxa are members of the class Clostridia and the class Bacilli. At day 0, members of the order Clostridiales reached the maximum LDA score of 4.67 and the genus *Anaerococcus* the lowest score of 3.07 (Fig. 4A); the first one is reported to have crucial functions in thermophilic lignocellulose decomposition (Strang et al. 2017), while the last one is capable of metabolizing peptones and amino acids, being the major metabolic end-products, butyric acid, lactic acid, and small quantities of propionic and succinic acids (Ezaki et al. 2001). The order Bacillales, is another increased abundant taxon that is reported augmented in mesophilic anaerobic digestion of corn stalks (Yu et al. 2019). On day 17 the order Clostridiales BSA2B-08 had a value of 3.62, while members of the orders Tenericutes, RF3, ML615J-28 had a value of 3.27 (Fig. 4A); the first one is reported as syntrophic acetate- and butyrate-oxidizing bacteria (Jiang et al. 2019), although for the second one there are no reports related to anaerobic digestion, it is reported that abundance of members of this taxon is enhanced by consumption of short-chain galacto-oligosaccharides in lactose intolerant humans (Azcarate-Peril et al. 2017); and finally at day-32, the family D2 of the clostridial order SHA_98 had a value of 4.35 while the family ML1228J-1 of the order Natranaerobiales, a value of 3.73 (Fig. 4A). It is reported that the clostridial family D2 had a negative correlation with propionate, during dry co-digestion of food waste and pig manure (Jiang et al. 2019), while the family ML1228J-1, has been reported in solid state anaerobic digestion of corn stover (Li et al. 2016). The rest of the bacteria include taxa reported in diverse mesophilic and thermophilic anaerobic digestion processes (Table 1).

**Table 1:** Significant abundant bacteria in MC50 treatment

Axa	This work	Other reports	References
Phylum firmicutes			
o_Clostridiales	Day-0 > fourfold	Perform crucial function in thermophilic lignocellulose decomposition	Strang et al. (2017)
g_*Defluviitalea*	Day-0 > fourfold	Thermophilic anaerobic saccharolytic bacterium isolated from AD treating animal manure and rice straw	Ma et al. (2017)
		Displayed optimal growth parameters at thermophilic conditions and was able to metabolize cellobiose, as well as acetate	Kinet et al. (2015)
g_*Tepidimicrobium*	Day-0 > fourfold	It was found at higher relative abundance in thermophilic AD compared to mesophilic. Members of this genus can utilize carbohydrates and proteinaceous compounds to produce VFAs	Wu et al. (2020)
o_Bacillales	Day-0 > threefold	Lignocellulose degradation increased as populations of Bacillales augmented in mesophilic anaerobic digestion of corn stalks	Jiang et al. (2019)
f_Haloplasmataceae	Day-0 > threefold	Increased up to two orders of magnitude in abundance during digestate recirculation in batch systems of yard trimmings. They have cellulolytic ability	Lin and Li (2017)
f_Bacillaceae	Day-0 > threefold	Members of this family are involved in hydrolysis, acidogenesis and acetogenesis	Oladejo et al. (2020)
c_Bacilli	Day-0 > threefold	Aerobic and anaerobic bacteria belonging to Bacilli were vastly found in substrates (food waste, cow dung and piggery dung), fermenting mixtures and digestate, these bacteria are involved in hydrolysis, acidogenesis and acetogenesis	Oladejo et al. (2020)
f_Tissierellaceae	Day-0 > threefold	*Caldicoprobacter* and *Tepidimicrobium* (family Tissierellaceae) were the predominant genera in thermophilic AD process with digestate recirculation. Both are fermentative microorganisms	Zamanzadeh et al. (2016)
g_*Anaerococcus*	Day-0 > threefold	This genus can metabolize peptones and amino acids and the major metabolic end-products are butyric acid, lactic acid, and small amounts of propionic and succinic acids	Yu et al. (2019)
c_Clostridia; o_BSA2B_08	Day-17 > threefold	It has not previously been reported in anaerobic digestion systems however, some members can act as syntrophic acetate- and butyrate-oxidizing bacteria	Azcarate-Peril et al. (2017)
f_Peptococcaceae	Day-17 > threefold	Bacterial family that could participate in direct interspecies electron transferred mediated syntrophic process with methanogens	Wu et al. (2019)
		Syntrophic obligate propionate oxidizers found in digesters with ammonia and zeolite. Propionate degradation occurred earlier in this condition in comparison with ammonia and no zeolite	Cardona et al. (2021)
g_*Syntrophomonas*	Day-17 > threefold	Propionate degradation was enhanced in bioaugmented reactor by *Syntrophomonas*	Lianhua et al. (2018)
g_*Lutispora*	Day-17 > threefold	Enriched at thermophilic conditions, and function as syntrophic acetate oxidation, syntrophic alcohol and lactate degradation and proteinaceous degradation	Jiang et al. (2020)
o_Natranaerobiales; f_ML1228J_1	Day-32 > threefold	This family has been reported in solid state anaerobic digestion of corn stover with methane production	Li et al. (2016)
c_Clostridia; o_SHA_98; f_D2	Day-32 > fourfold	During dry co-digestion of food waste and pig manure it was found that this family had a negative correlation with propionate	Jiang et al. (2019)
g_*Pelotomaculum*	Day-32 > threefold	Syntrophic propionate oxidizers can be found within this genus	Tsapekos et al. (2017)
Phylum tenericutes			
c_RF3; o_ML615J_28	Day-17 > threefold	The abundance of members of this taxon enhanced by consumption of short-chain galacto-oligosaccharides in lactose intolerant humans	Azcarate-Peril et al. (2017)

e.g., day-0 > fourfold, indicates fourfold more abundant at day 0; o_order; g_genera; f_family; c_class

The LefSe analysis did not show a significant change in the abundance of archaeal taxa, however one-way ANOVA analysis showed that three archaeal taxa the order E2 (class Thermoplasmata), family Methanomicrobiaceae, and *Methanoculleus bourgensis* were significantly different between day-0 and days 17 and 32 (Fig. 4B). A pure culture of *Methanoculleus bourgensis* was used in a reported bioaugmentation process to overcome ammonia inhibition due to AD, resulting in 28% increase methane yield (Tian et al. 2019).

**Fig. 5:**
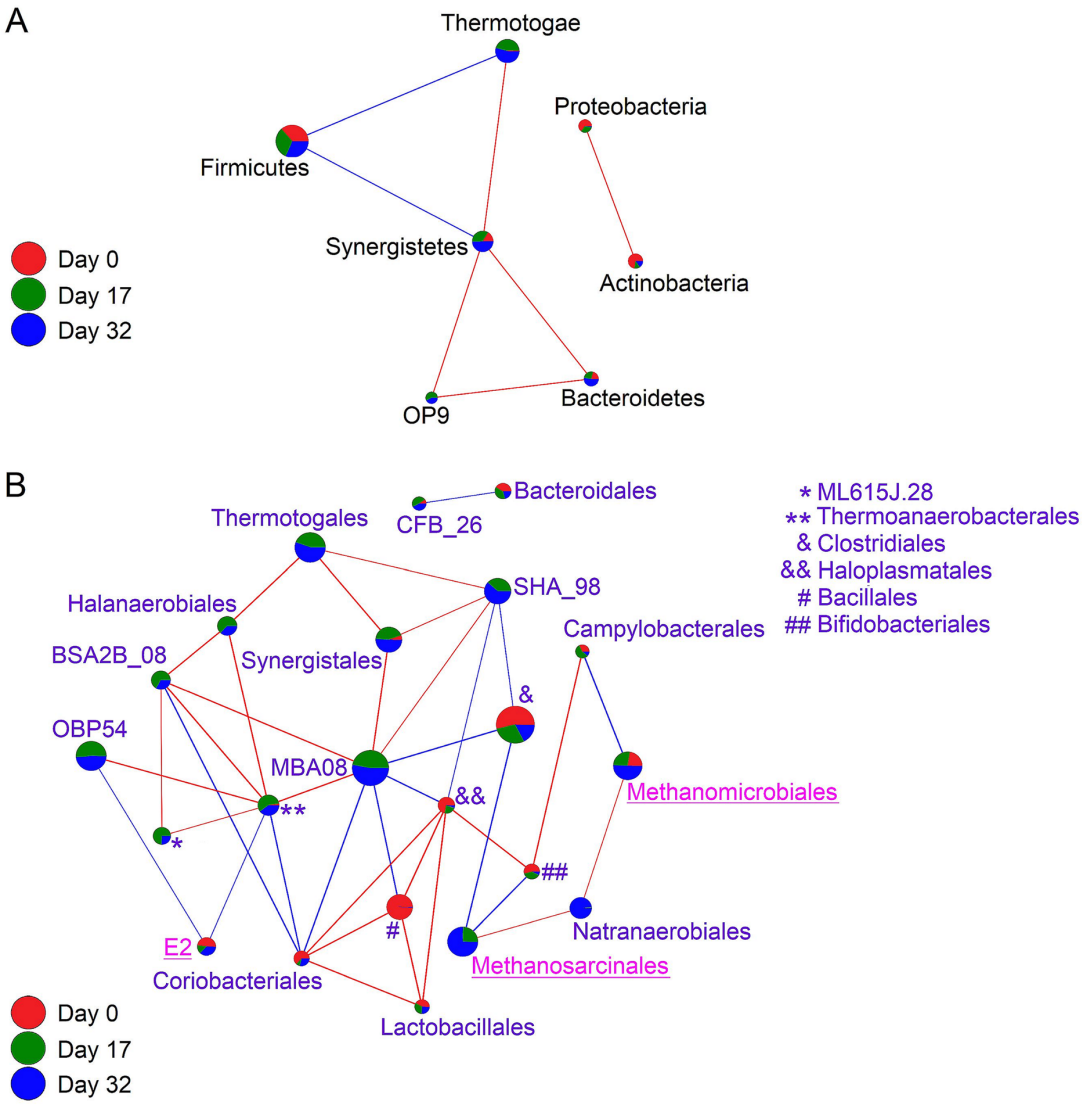
Co-occurrence network for bacteria at the phylum level (**a**), and bacteria-archaeal at the order level (**b**). In the circled nodes, the size of the color sectors for each phylum represents the abundance at each time; the size of the circles is in proportion to the number of connections to each phylum. Blue color lines indicate negative correlations, red color lines indicate positive correlations. Day 0 (red), day 17 (green), and day 32 (blue), tags are located on the bottom left side

Microorganisms in a consortium sustain positive and/or negative interactions with other members. A correlation network analysis of interactions for the bacterial phyla in the MC50 reactor revealed that the phylum Firmicutes had a negative correlation with members of the phyla Thermotogae and Synergistetes, while the last two had a positive correlation between them. The same analysis showed that the phyla OP9, Bacteroidetes, and Synergistetes had a positive correlation among them. On the other hand, the phyla Proteobacteria and Actinobacteria had a separate positive correlation between them (Fig. 5A). Same analysis for the archaeal phyla in the MC50 reactor revealed only one significant negative interaction between the genera *Methanosarcina* and *Methanoculleus* (results not shown). This can be explained since each of them belong to a different methane generation pathway.

When simultaneous interactions between members of the bacterial and archaeal communities were evaluated at the order level, the archaea Methanomicrobiales showed a positive correlation with the bacterial order Campylobacterales, and a negative correlation with the bacteria Natranaerobiales. In addition, the archaea Methanosarcinales, showed a positive correlation with the bacteria Natranaerobiales, and a negative correlation with the bacterial orders Bifidobacteriales and Clostridiales. The archaeal order E2, showed two negative correlations with the bacteria OPB54 and Thermoanaerobacterales. The bacterial order Thermoanaerobacterales showed seven interactions, two negative correlations with the archaea E2 and bacteria Coriobacteriales, and five positive correlations with the bacterial orders ML615J_28, OPB54, BSA2B_8, Halanaerobiales, and MBA08. Finally, the bacterial order MBA08 showed eight interactions, four negative correlations with the bacterial orders Clostridiales, Haloplasmalales, Bacillales, Coriobacteriales, and four positive correlations with the bacteria Thermoanaerobacterales, BSA2B_8, Synergistales and SHA_98 (Fig. 5B).

### Relevant metabolic pathways for substrate degradation and methane production

A prediction of the metabolic pathways in the consortium based on the taxa abundance showed 404 pathways for the MC50 treatment in bacteria and 263 for archaea. From these only 51 had significant statistical differences in abundance for bacteria and 15 for archaea at days 0, 17, and 32 (Table S5). The pathways were grouped into the ones that increased from day 0 to day 32, such as flavin biosynthesis, L-arginine biosynthesis, L-ornithine biosynthesis, pentose phosphate pathway, and coenzyme B biosynthesis (archaea). Coenzyme B is exclusive for methanogens and acts as an electron carrier, required for methane formation (Thauer 2019). The pathways that decreased from day 0 to day 32, glycolysis, homolactic fermentation, purine nucleobases degradation, superpathway of demethylmenaquinol-8 biosynthesis, and superpathway of hexitol degradation; and the ones that increased at day 17 and decreased by day 32: 8-amino-7-oxononanoate biosynthesis and methanol oxidation to carbon dioxide (Fig. S7A-L). Some of these pathways were also found at thermophilic conditions in an up-flow anaerobic reactor (Liang et al. 2021).

## Conclusion

In this work, we characterized the diversity of bacterial and archaeal microbial communities engaged in a bioaugmentation technique to improve the methane yield of a thermophilic AD process. The content of hydrogenotrophic methanogens was enriched and reintegrated into the system as a methanogenic consortium. We determined that the MC was enriched with bacteria and methanogens whose activity increased the methane yield up to 4% during OFMSW degradation. Significant changes in the relative abundance of taxa and microbial interactions were observed where an increase in the archaeal hydrogenotrophic *Methanoculleus* and the bacterial Clostridia order MBA08 when using 50% of consortium. Predicted relevant metabolic pathways confirmed the substrate degradation and the anaerobic methanogenic process.

## Supplementary Information

Below is the link to the electronic supplementary material.

Supplementary file1 (PDF 1777 KB)

## Data Availability

The sequencing data for bacteria and archaea from this research are available through the NCBI database under the project accession number PRJNA630827 or the following link: https://www.ncbi.nlm.nih.gov/bioproject/PRJNA630827.
