# Supplementary material for: Monitoring of a microbial community during bioaugmentation with hydrogenotrophic methanogens to improve methane yield of an anaerobic digestion process
Source: Biotechnol Lett. 2023 Aug 3;45(10):1339–53. doi: 10.1007/s10529-023-03414-7 (PMC10460350; doi:10.1007/s10529-023-03414-7)
Supplement: Supplementary file 1 — Supplementary file1 (PDF 1777 KB) [file 10529_2023_3414_MOESM1_ESM.pdf]

Supplementary Material

Monitoring of a microbial community during bioaugmentation with hydrogenotrophic methanogens to improve methane yield of an anaerobic digestion process

Aixa Kari Gállego Bravo, Jaime García-Mena, Alberto Piña-Escobedo, Gloria López Jiménez, María Eugenia Gutiérrez Castillo, and Luis Raúl Tovar Gálvez.

Tables

Table S1. Physicochemical characteristics of the digestate.

Table S2. Sequencing summary for total Samples.

Table S3. Statistics for Alpha diversity indexes.

Table S4. Statistics for Beta diversity.

Table S5. Selected metabolic pathways for the MC_50 treatment.

Figures

Figure S1. Rarefaction curves showing richness based on observed OTUs at 99% similarity.

Figure S2. Relative abundance of predominant phyla in Organic Fraction of Municipal Solid Waste (OFMSW), and Methanogenic Consortium (MC).

Figure S3. Relative abundance of predominant taxa in Organic Fraction of Municipal Solid Waste (OFMSW), and Methanogenic Consortium (MC).

Figure S4. Relative abundance of predominant bacterial phyla and archaeal class in batch thermophilic anaerobic digesters.

Figure S5. Microbial diversity during the thermophilic anaerobic digestion over time, average.

Figure S6. Microbial diversity during the thermophilic anaerobic digestion over time, replicas.

Figure S7. Comparative prediction of the functional metagenome for the MC50 treatment.

Table S1. Physicochemical characteristics of the digestate.

	Parameters						
**Treatments**	**pH**	**ORP (mV)**	**EC (mS/cm)**	**TS (g/kg)**	**VS (g/kg)**	**VS/TS ratio (%)**	**VS reduction (%)**
MC00	8.45 ± 0.03	-83.70 ± 0.90	51.15 ± 6.28	22.54 ± 4.66	7.71 ± 0.83	34.79 ± 5.32	63.12 ± 8.63
MC10	8.20 ± 0.10	-33.17 ± 3.35	46.29 ± 4.97	17.27 ± 1.50	4.06 ± 0.45	23.51 ± 0.69	72.50 ± 1.06
MC25	8.21 ± 0.11	-21.23 ± 1.45	38.12 ± 5.29	21.45 ± 1.22	5.04 ± 0.33	23.50 ± 1.41	67.95 ± 6.30
MC50	7.91 ± 0.07	-5.45 ± 1.25	30.18 ± 1.06	12.19 ± 1.10	2.98 ± 0.30	24.42 ± 0.26	71.10 ± 0.41
MC75	7.82 ± 0.04	5.40 ± 1.20	22.71 ± 3.00	7.25 ± 1.04	1.67 ± 0.28	22.92 ± 0.78	73.38 ± 1.17
p value	<0.001[a]	0.009[b]	<0.001[a]	<0.001[a]	0.009[b]	0.057[b]	0.138[a]

ORP: oxidation-reduction potential; EC: electrical conductivity; TS: total solids; VS: volatile solids. MC00 (inoculum + OFMSW), MC10 (90% inoculum + 10% MC v/v + OFMSW), MC25 (75% inoculum + 25% MC v/v + OFMSW), MC50 (50% inoculum + 50% MC v/v + OFMSW), and MC75 (25% inoculum + 75% MC v/v + OFMSW). a: One-way ANOVA. b: Kruskal-Wallis One-way ANOVA.

Table S2. Sequencing summary for total Samples.

Parameter	Bioaugmentation experiment N=54	Methanogenic consortium N=1	OFMSW N=1		Total N=56
	**Bacterial**				
Number of raw reads	3,272,109	101,845	40,263		3,414,217
Number of reads after quality filtering	2,433,399	76,204	30,715		2,540,318
Length mean[a]	160	160	160		160
Reads mean	45,062.94	N.A.	N.A.		45,362.82
Reads min–max	14,706-101,920	N.A.	N.A.		14,706-101,920
Total frequency of features[b]	2,433,399	76,204	30,715	6,818[c]	2,516,421
Number of identified features	1,576	101	155	147[c]	1,696
Samples with <14,000 reads	0	0	0	0	0
	**Bioaugmentation experiment N=50**	**Methanogenic consortium N=1**	**OFMSW N=0**		**Total N=51**
	**Archaeal**				
Number of raw reads	1,552,985	12,996	N.A.		1,565,981
Number of reads after quality filtering	904,273	9,941	N.A.		914,214
Length mean[a]	240	240	N.A.		240
Reads mean	11,179.92	N.A.	N.A.		11,031.57
Reads min–max	1,051-33,556	N.A.	N.A.		1,051-33,556
Total frequency of features[b]	558,996[d]	3,614[d]	N.A.	N.A.	562,610
Number of identified features	308[d]	19[d]	N.A.	N.A.	314
Samples with <10,000 reads	25	1	N.A.	N.A.	26

[a]Length expressed as bases. [b]Features correspond to OTUs in QIIME2. [c]Filtered features, after data for phylum Cyanobacteria, class Chloroplast, order Streptophyta were removed from table.qza and taxonomy.qza files. [d]Filtered features, after data for kingdom Bacteria were removed from table.qza and taxonomy.qza files.

Table S3. Statistics for Alpha diversity indexes

Group	Days	Alpha diversity							
		Bacteria				Archaea			
		Observed	Chao1	Shannon	Simpson	Observed	Chao1	Shannon	Simpson
**AI100**	Day 0	418 ± 74.64	418 ± 74.64	4.49 ± 0.01	0.94 ± 0.01	33.33 ± 4.99	33.33 ± 4.99	2.82 ± 0.21	0.91 ± 0.02
	Day 17	412 ± 19.01	412 ± 19.01	4.11 ± 0.10	0.90 ± 0.01	54.50 ± 3.50	54.50 ± 3.50	3.77 ± 0.07	0.97 ± 0.002
	Day 32	329 ± 47.75	329 ± 47.75	3.87 ± 0.14	0.90 ± 0.004	55.67 ± 2.49	55.67 ± 2.49	3.66 ± 0.23	0.97 ± 0.01
	p-value	0.242[a]	0.242[a]	0.002[a]	0.006[a]	0.005[a]	0.005[a]	0.011[a]	0.013[a]
**MC_00**	Day 0	254 ± 30.38	254 ± 30.38	3.35 ± 0.31	0.89 ± 0.05	40.33 ± 8.96	40.33 ± 8.96	3.06 ± 0.30	0.93 ± 0.02
	Day 17	181 ± 7.79	181 ± 7.79	2.84 ± 0.17	0.86 ± 0.04	17.50 ± 11.50	17.50 ± 11.50	2.16 ± 0.45	0.85 ± 0.05
	Day 32	133 ± 2.87	133 ± 2.87	2.90 ± 0.17	0.89 ± 0.01	25.33 ± 4.03	25.33 ± 4.03	2.13 ± 0.16	0.85 ± 0.01
	p-value	0.002[a]	0.002[a]	0.124[a]	0.690[a]	0.132[a]	0.132[a]	0.030[a]	0.075[b]
**MC_10**	Day 0	146 ± 5.72	146 ± 5.72	2.52 ± 0.59	0.76 ± 0.12	35.00 ± 7.79	35.00 ± 7.79	3.04 ± 0.16	0.94 ± 0.01
	Day 17	151 ± 27.19	151 ± 27.19	2.59 ± 0.22	0.79 ± 0.07	25.33 ± 3.30	25.33 ± 3.30	2.69 ± 0.26	0.91 ± 0.03
	Day 32	147 ± 20.24	147 ± 20.24	2.69 ± 0.14	0.85 ± 0.01	31.33 ± 4.78	31.33 ± 4.78	2.65 ± 0.29	0.90 ± 0.03
	p-value	0.932[a]	0.932[a]	0.895[a]	0.557[a]	0.294[a]	0.294[a]	0.280[a]	0.379[a]
**MC_25**	Day 0	161 ± 49.20	161 ± 49.20	2.84 ± 0.26	0.86 ± 0.06	21.67 ± 6.34	21.67 ± 6.34	2.56 ± 0.32	0.90 ± 0.04
	Day 17	124 ± 17.75	124 ± 17.75	2.64 ± 0.35	0.81 ± 0.09	18.00 ± 1.63	18.00 ± 1.63	2.56 ± 0.10	0.91 ± 0.01
	Day 32	142 ± 38	142 ± 38	2.99 ± 0.07	0.88 ± 0.02	27.00 ± 7.79	27.00 ± 7.79	2.51 ± 0.28	0.89 ± 0.03
	p-value	0.634[a]	0.634[a]	0.424[a]	0.486[a]	0.368[a]	0.368[a]	0.973[a]	0.814[a]
**MC_50**	Day 0	130 ± 35.52	130 ± 35.52	3.19 ± 0.15	0.92 ± 0.01	18.50 ± 1.50	18.50 ± 1.50	2.61 ± 0.05	0.91 ± 0.01
	Day 17	216 ± 3.68	216 ± 3.68	3.22 ± 0.08	0.91 ± 0.01	19.00 ± 3.27	19.00 ± 3.27	2.47 ± 0.08	0.89 ± 0.02
	Day 32	124 ± 6.02	124 ± 6.02	2.87 ± 0.20	0.87 ± 0.02	16.67 ± 0.47	16.67 ± 0.47	2.40 ± 0.12	0.88 ± 0.02
	p-value	0.005[a]	0.005[a]	0.102[a]	0.068[a]	0.600[b]	0.600[b]	0.224[a]	0.390[a]
**MC_75**	Day 0	111 ± 14.70	111 ± 14.70	2.84 ± 0.19	0.88 ± 0.03	15.50 ± 1.50	15.50 ± 1.50	2.34 ± 0.32	0.88 ± 0.04
	Day 17	130 ± 41.61	130 ± 41.61	2.80 ± 0.28	0.86 ± 0.05	21.33 ± 2.62	21.33 ± 2.62	2.40 ± 0.36	0.88 ± 0.05
	Day 32	116 ± 9.10	116 ± 9.10	2.60 ± 0.14	0.83 ± 0.02	26.67 ± 7.32	26.67 ± 7.32	2.71 ± 0.11	0.92 ± 0.004
	p-value	0.749[a]	0.749[a]	0.505[a]	0.410[a]	0.226[a]	0.226[a]	0.470[a]	0.572[a]
p-value (groups)*		<0.001[a]	<0.001[a]	<0.001[a]	0.004[b]	<0.001[a]	<0.001[a]	0.004[b]	0.030[b]

a) One-Way ANOVA, b) Kruskal-Wallis. * Statistical analysis among all treatment groups.

Table S4. Statistics for Beta diversity

Group 1	Group 2	Beta diversity			
		Bacteria		Archaea	
		***p*-value**	***q*-value**	***p*-value**	***q*-value**
AI100	MC_00	0.001	0.002	0.021	0.063
	MC_10	0.001	0.002	0.021	0.063
	MC_25	0.001	0.002	0.003	0.023
	MC_50	0.001	0.002	0.001	0.015
	MC_75	0.001	0.002	0.010	0.050
MC_00	MC_10	0.180	0.194	0.917	0.917
	MC_25	0.013	0.019	0.727	0.779
	MC_50	0.005	0.009	0.058	0.124
	MC_75	0.001	0.002	0.660	0.779
MC_10	MC_25	0.181	0.194	0.694	0.779
	MC_50	0.034	0.043	0.097	0.178
	MC_75	0.001	0.002	0.716	0.779
MC_25	MC_50	0.248	0.248	0.107	0.178
	MC_75	0.014	0.019	0.637	0.779
MC_50	MC_75	0.014	0.019	0.058	0.124

Table S5. Selected metabolic pathways for the MC_50 treatment.

Bacteria			Archaea		
**Pathway**	***p*-value**	***q*-value**	**Pathway***	***p*-value**	***q*-value**
8-amino-7-oxononanoate biosynthesis I	<0.001	0.008	Adenosine nucleotides degradation IV	0.027	--
Flavin biosynthesis I	<0.001	0.008	Coenzyme A byosynthesis	0.047	
Glycolysis I (from glucose 6-phosphate)	0.001	0.018	Coenzyme B biosynthesis	0.026	--
Homolactic fermentation	<0.001	0.009	Formaldehyde assimilation II (RuMP Cycle)	0.018	--
L-arginine biosynthesis II (acetyl cycle)	<0.001	0.004	Glycolysis I (from glucose 6-phosphate)	0.005	--
L-ornithine biosynthesis	<0.001	0.009	Glycolysis II (from fructose 6-phosphate)	0.004	--
Methanol oxidation to carbon dioxide	0.004	0.039	Glycolysis III (from glucose)	0.001	--
Pentose phosphate pathway	0.001	0.019	L-arginine biosynthesis II (acetyl cycle)	0.041	--
Purine nucleobases degradation I (anaerobic)	0.004	0.041	L-arginine biosynthesis III (via N-acetyl-L-citrulline)	0.022	--
Superpathway of demethylmenaquinol-8 biosynthesis	<0.001	0.010	L-lysine biosynthesis I	0.037	--
Superpathway of hexitol degradation	0.005	0.045	L-methionine biosynthesis III	0.030	--
			L-ornithine biosynthesis	0.024	--
			Mixed acid fermentation	0.008	--
			O-antigen building blocks biosynthesis	0.038	--
			thiamin salvage II	0.034	

Data for 9 samples (n=9); *: Without Benjamini-Hochberg correction.

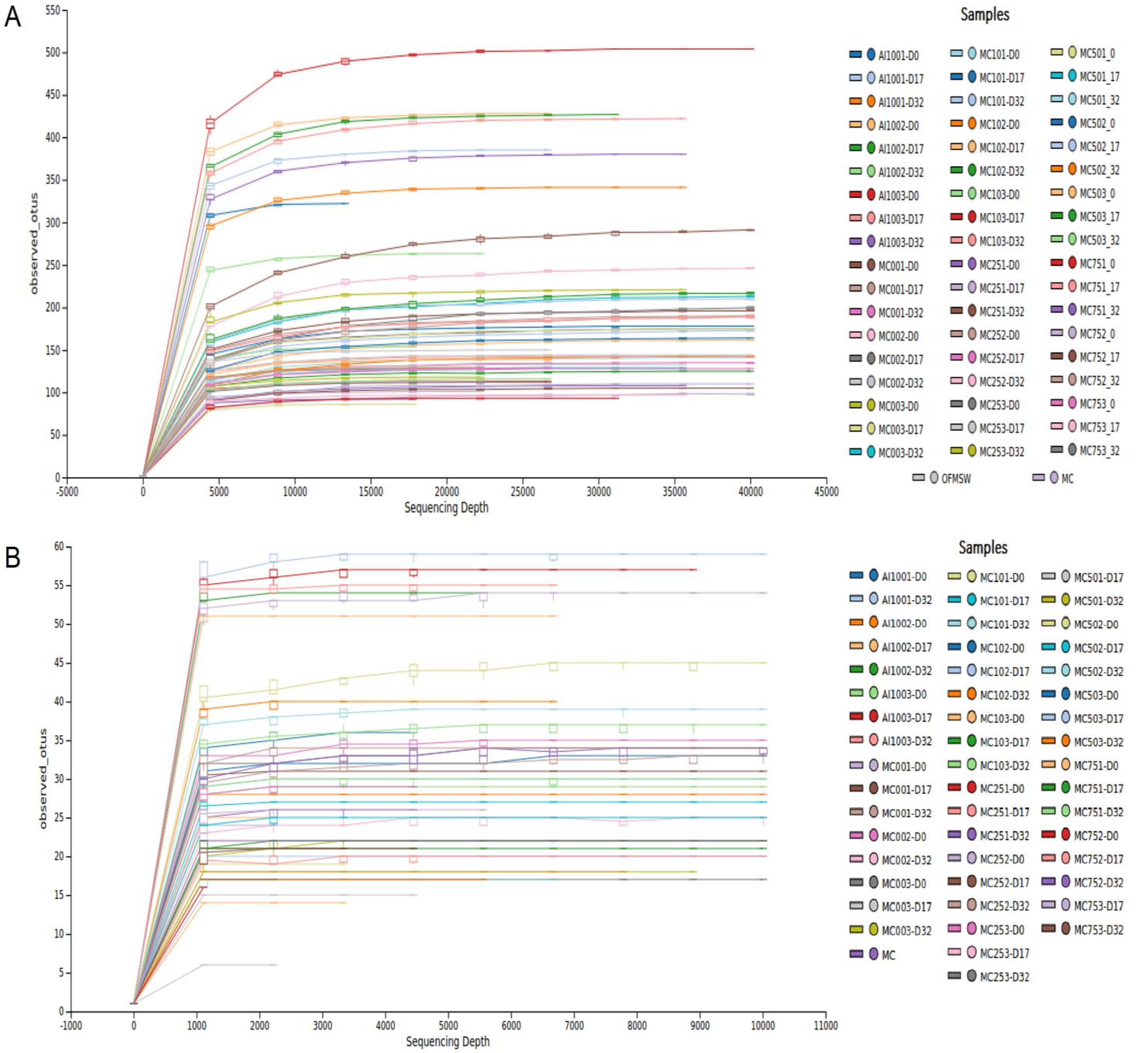

Figure S1. Rarefaction curves showing richness based on observed OTUs at 99% similarity. Plots show total samples identified as AI100 (Acclimated Inoculum at 100%), MC00, MC10, MC25, MC50 and MC75 (Acclimated Inoculum + OFMSW + MC at 0, 10, 25, 50 and 75%). The following digit indicates the number of replica in the test (1–3), and the characters after "-D" indicate the sampling day (0, 17, 32). OFMSW (Organic Fraction of Municipal Solid Waste), and MC (Methanogenic Consortium). (A) Bacteria, (B) Archaea.

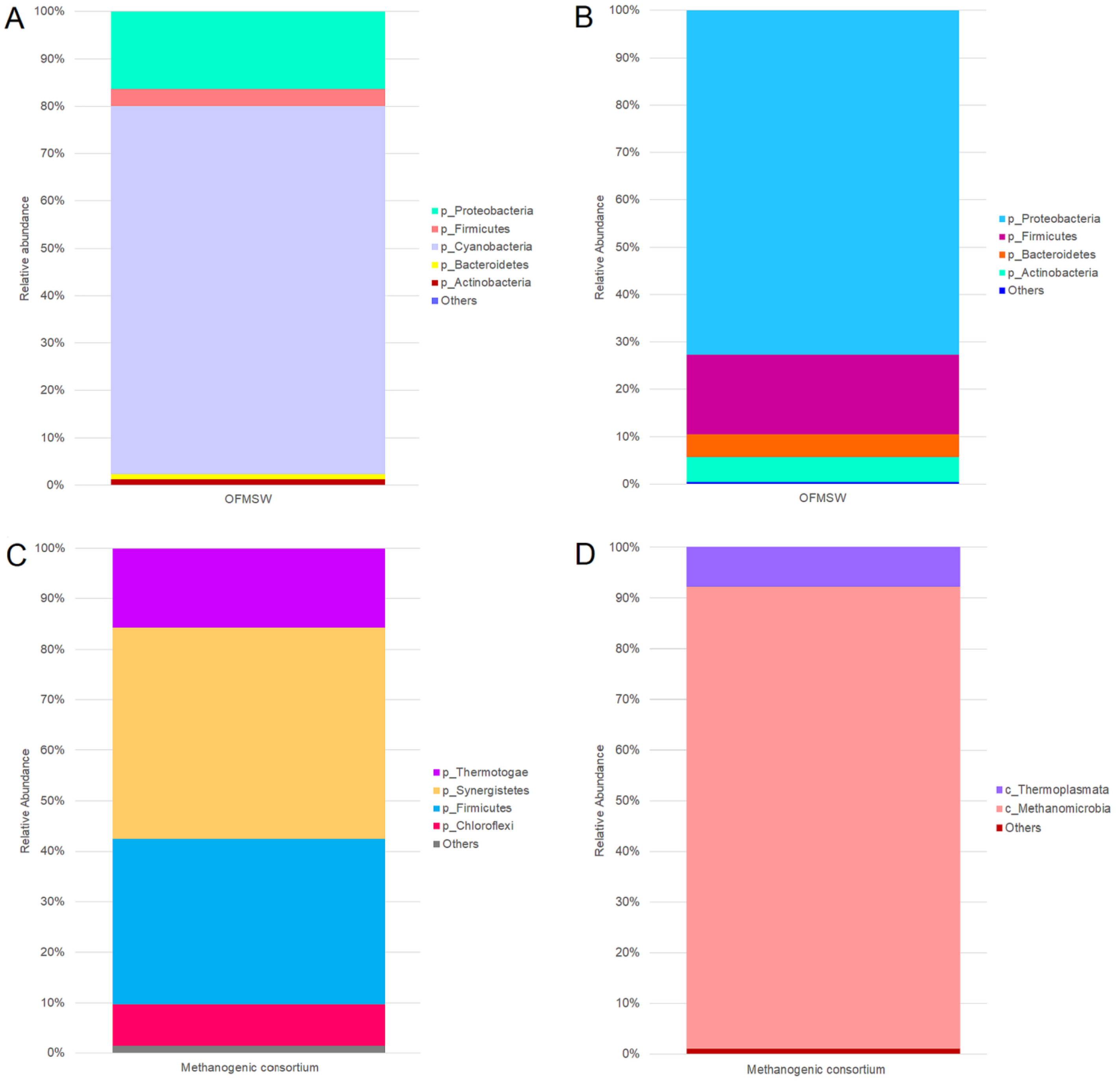

Figure S2. Relative abundance of predominant phyla in Organic Fraction of Municipal Solid Waste (OFMSW), and Methanogenic Consortium (MC). The stacked bar charts show abundances for Unfiltered sequences for Streptophyta (Cyanobateria phylum) of OFMSW for bacteria (A); Filtered sequences for Streptophyta (Cyanobateria phylum) of OFMSW for bacteria (B); and Sequences of Methanogenic Consortium (MC) for bacteria (C), and archaea (D). Y-axis indicates the percentage of relative abundance; X-axis indicates the sample. Tags at the right side of each set of graphic bars identify the corresponding taxa by color.

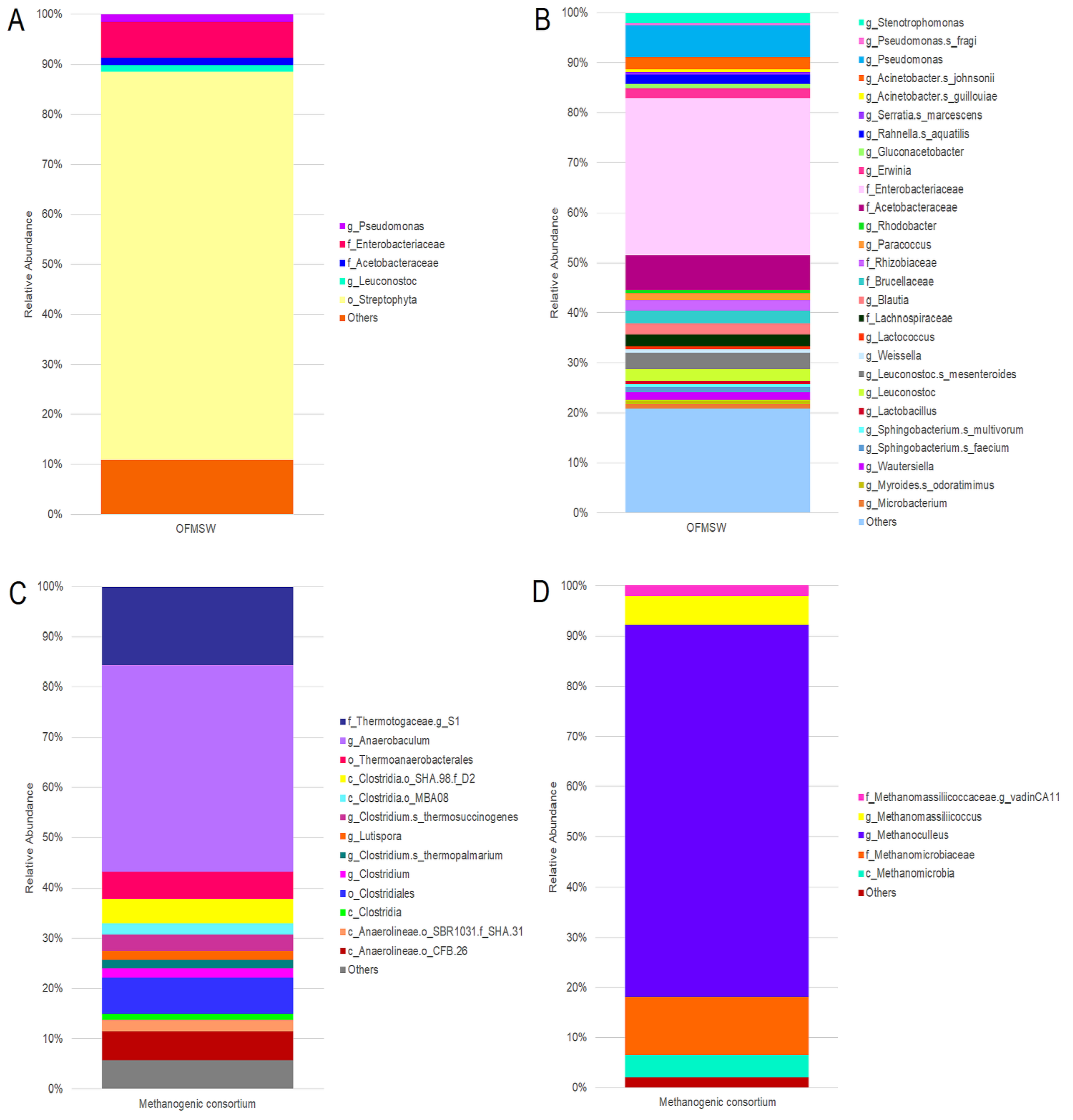

Figure S3. Relative abundance of predominant taxa in Organic Fraction of Municipal Solid Waste (OFMSW), and Methanogenic Consortium (MC). The stacked bar charts show abundances for Unfiltered sequences for Streptophyta (Cyanobateria phylum) of OFMSW for bacteria (A); Filtered sequences for Streptophyta (Cyanobateria phylum) of OFMSW for bacteria (B); and Sequences of Methanogenic Consortium (MC) for bacteria (C), and archaea (D). Y-axis indicates the percentage of relative abundance; X-axis indicates the sample. Tags at the right side of each set of graphic bars identify the corresponding taxa by color.

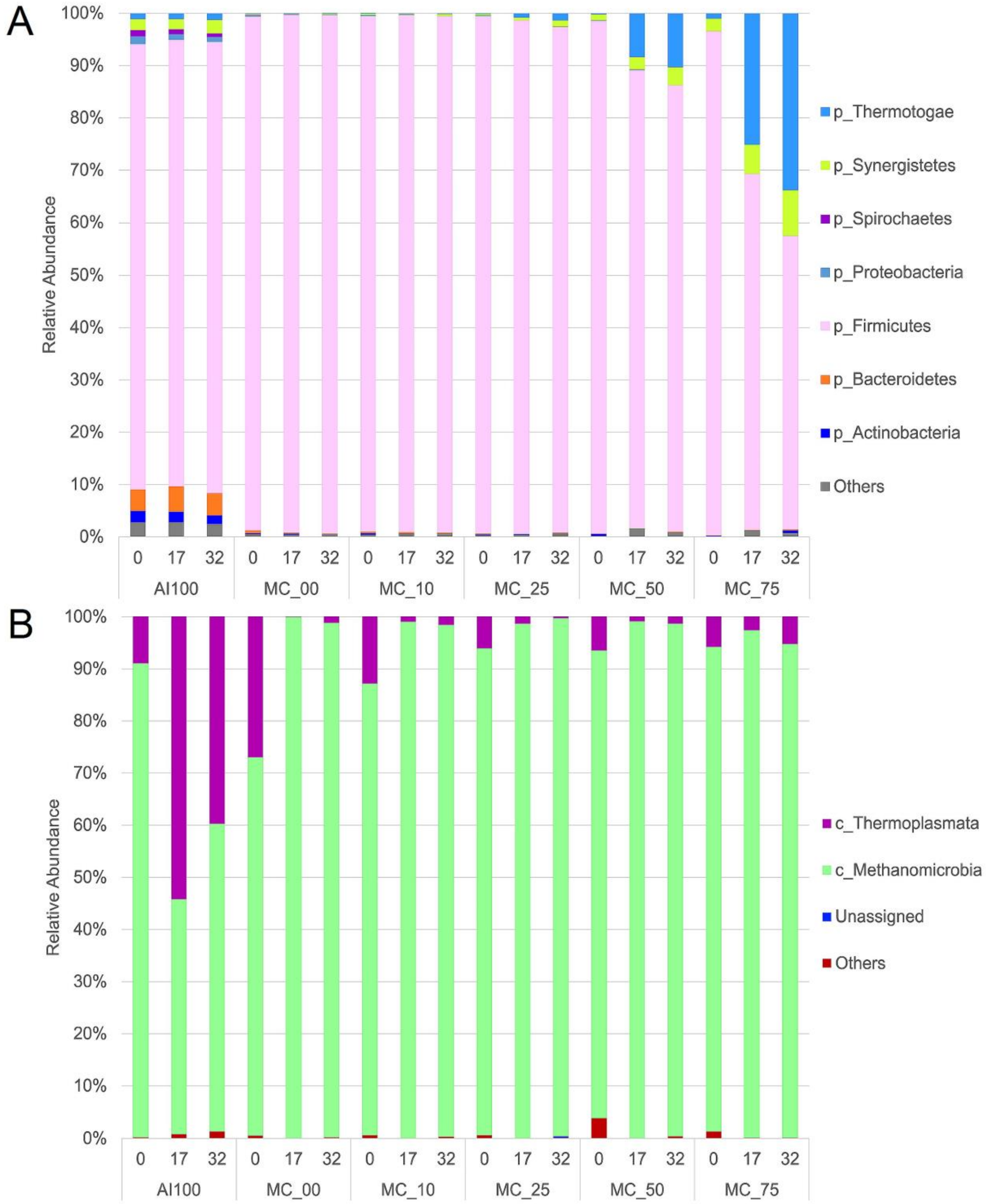

Figure S4. Relative abundance of predominant bacterial and archaeal taxa in batch thermophilic anaerobic digesters. The stacked bar charts show abundances for (a) bacterial phyla, (b) archaeal class, for the methanogenic consortium at 0% (MC00), 10% (MC10), 25% (MC25); 50% (MC50), 75% (MC75), and the negative control for the process (AI100). Y-axis indicates the percentage of relative abundance; X-axis indicates time in days and treatment. Tags at the right side of each set of graphic bars identify the corresponding taxa by color. Plotted data are the average of three independent sequencing experiments.

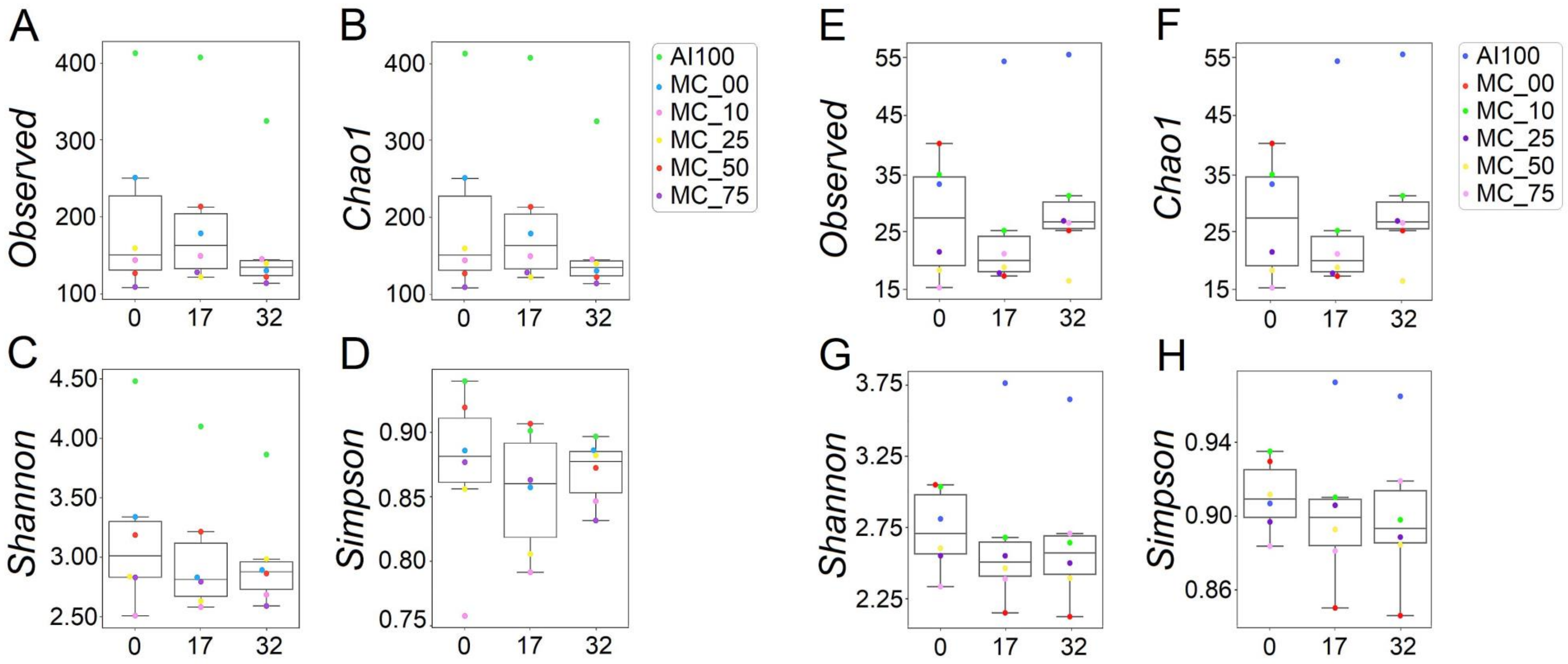

Figure S5. Microbiota diversity during the thermophilic anaerobic digestion over time. The top part of the figure shows box-plot graphs for alpha-diversity analyses of Observed species, bacteria (a), archaea (e); expected (Chao1) richness, bacteria (b), archaea (f); Shannon diversity, bacteria (c), archaea (g); and Simpson diversity, bacteria (d), archaea (h) indexes. One-way ANOVA and Kruskal-Wallis analyses were used to find significant differences. The Y-axis indicates the values for each index, the X-axis, indicates time in days (0, 17, and 32). Color tags at the upper right position beside each box-plot set identify each methanogenic consortium concentration 0% (MC00), 10% (MC10), 25% (MC25); 50% (MC50), 75% (MC75), and the negative control for the process (AI100). Plotted data are the average of three independent sequencing experiments.

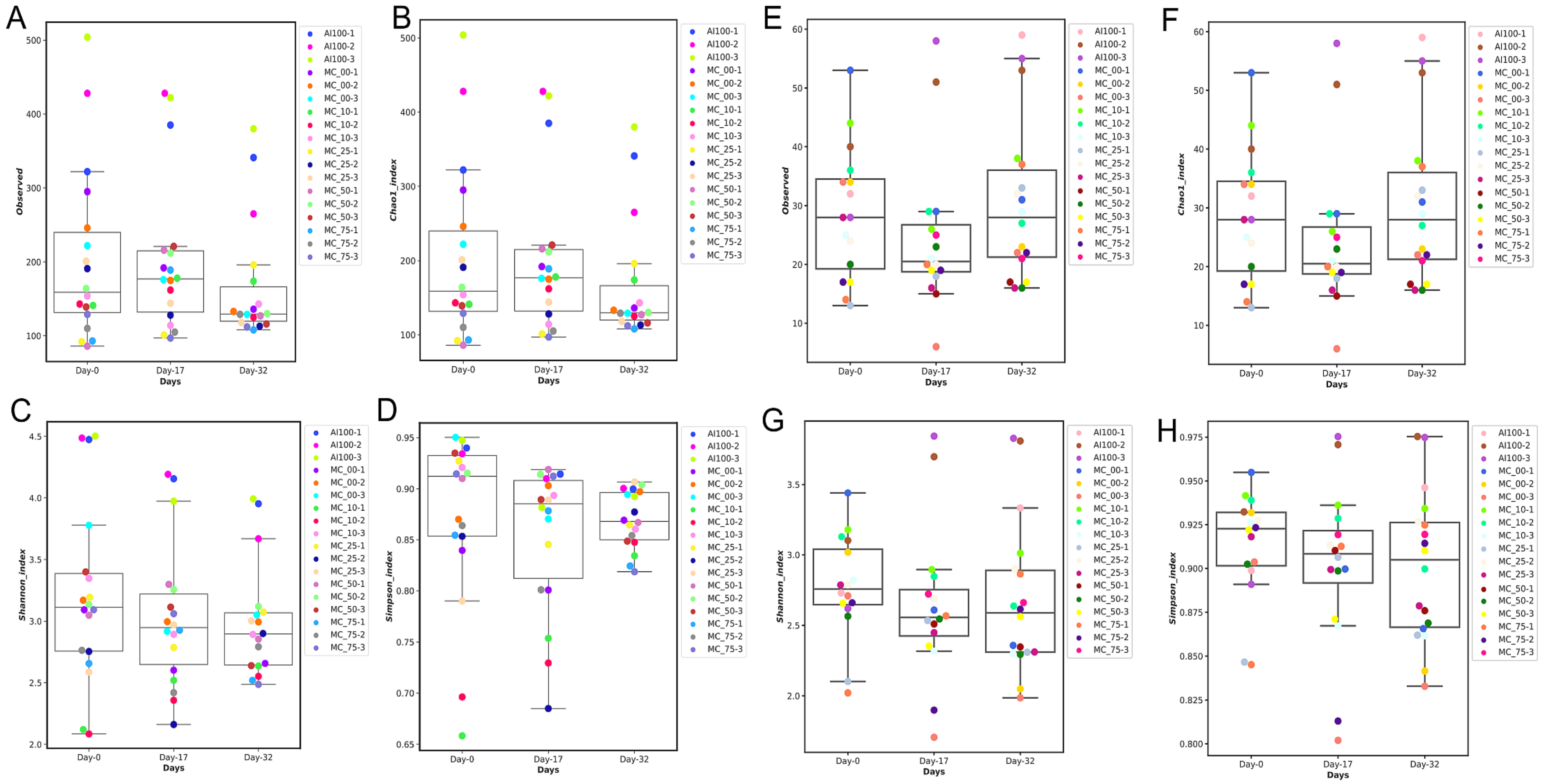

Figure S6. Microbial diversity during the thermophilic anaerobic digestion over time. The figure shows box-plot graphs for alpha-diversity analyses of Observed species for bacteria (A), archaea (E); expected (Chao1) richness for bacteria (B), and archaea (F); Shannon diversity for bacteria (C), and archaea (G); and Simpson diversity for bacteria (D) and archaea (H). One-way ANOVA and Kruskal-Wallis analyses were used to find significant differences. The Y-axis indicates the values for each index, the X-axis, indicates time in days (0, 17, and 32). Color tags at the upper right position beside each box-plot set identify each methanogenic consortium concentration 0% (MC_00), 10% (MC_10), 25% (MC_25); 50% (MC_50), 75% (MC_75), and the negative control for the process (AI100). The last digit in the tag, indicates the number of replica in the test (1–3).

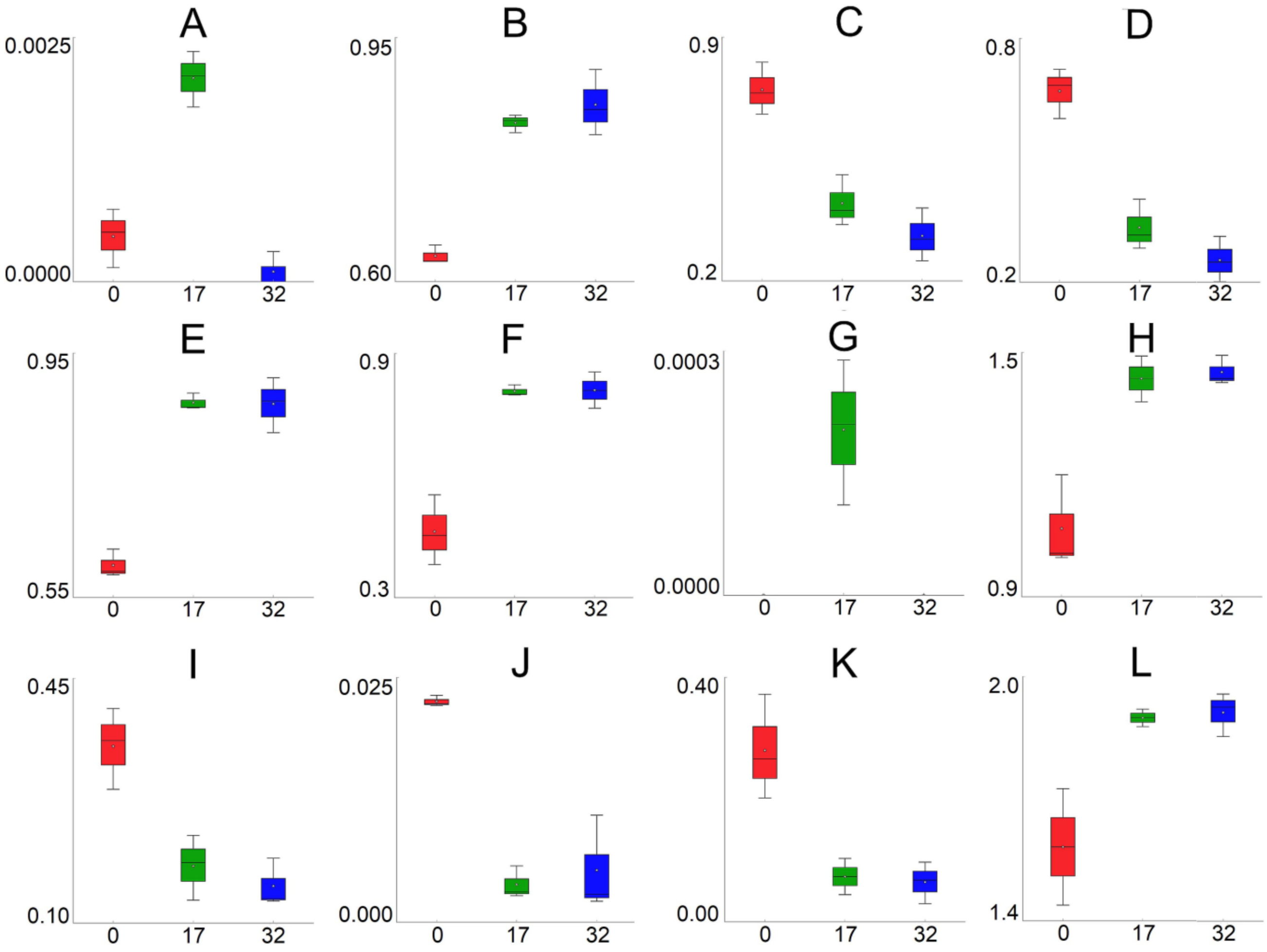

Figure S7. Comparative prediction of the functional metagenome for the MC50 treatment. The figure shows graphic boxplots representing the proportion of selected predicted metabolic pathways using PICRUSt2. The Y-axis shows the proportion of the predicted sequences, and the X-axis shows the time in days. (a) 8-amino-7-oxononanoate biosynthesis I, (b) Flavin biosynthesis I, (c) Glycolysis I, (d) Homolactic fermentation, (e) L-arginine biosynthesis II (acetyl cycle), (f) L-ornithine biosynthesis, (g) Methanol oxidation to carbon dioxide, (h) Pentose phosphate pathway, (i) Purine nucleobases degradation I (anaerobic), (j) Superpathway of demethylmenaquinol-8 biosynthesis, (k) Superpathway of hexitol degradation. The significant metabolic pathway shown at the bottom of the graph for archaea is: (l) Coenzyme B biosynthesis. The boxplots show the interquartile ranges (bottom and top of boxes), medians (middle lines in the boxes), mean (star symbol in the boxes) and the lowest and highest values for the first and third quartiles. Each time is identified by Day 0 (red), day 17 (green) and day 32 (blue).
